# Deep sequence modelling for predicting COVID-19 mRNA vaccine degradation

**DOI:** 10.7717/peerj-cs.597

**Published:** 2021-06-22

**Authors:** Talal S. Qaid, Hussein Mazaar, Mohammed S. Alqahtani, Abeer A. Raweh, Wafaa Alakwaa

**Affiliations:** 1 Computer Science Department, College of Computer Science, King Khalid University, Abha, Saudi Arabia; 2 Faculty of Computer Science, Hodeidah University, Hodeidah, Yemen; 3 Computer Science Department, College of Science & Arts in Tanumah, King Khalid University, Abha, Saudi Arabia; 4 Radiological Sciences Department, College of Applied Medical Sciences, King Khalid University, Abha, Saudi Arabia

**Keywords:** COVID-19 Vaccine, mRNA, Reccurent neural networks (RNN), Gated reccurent unit (GRU), Long short time memory (LSTM), Embedding

## Abstract

The worldwide coronavirus (COVID-19) pandemic made dramatic and rapid progress in the year 2020 and requires urgent global effort to accelerate the development of a vaccine to stop the daily infections and deaths. Several types of vaccine have been designed to teach the immune system how to fight off certain kinds of pathogens. mRNA vaccines are the most important candidate vaccines because of their capacity for rapid development, high potency, safe administration and potential for low-cost manufacture. mRNA vaccine acts by training the body to recognize and response to the proteins produced by disease-causing organisms such as viruses or bacteria. This type of vaccine is the fastest candidate to treat COVID-19 but it currently facing several limitations. In particular, it is a challenge to design stable mRNA molecules because of the inefficient in vivo delivery of mRNA, its tendency for spontaneous degradation and low protein expression levels. This work designed and implemented a sequence deep model based on bidirectional GRU and LSTM models applied on the Stanford COVID-19 mRNA vaccine dataset to predict the mRNA sequences responsible for degradation by predicting five reactivity values for every position in the sequence. Four of these values determine the likelihood of degradation with/without magnesium at high pH (pH 10) and high temperature (50 degrees Celsius) and the fifth reactivity value is used to determine the likely secondary structure of the RNA sample. The model relies on two types of features, namely numerical and categorical features, where the categorical features are extracted from the mRNA sequences, structure and predicted loop. These features are represented and encoded by numbers, and then, the features are extracted using embedding layer learning. There are five numerical features depending on the likelihood for each pair of nucleotides in the RNA. The model gives promising results because it predicts the five reactivity values with a validation mean columnwise root mean square error (MCRMSE) of 0.125 using LSTM model with augmentation and the codon encoding method. Codon encoding outperforms Base encoding in MCRMSE validation error using the LSTM model meanwhile Base encoding outperforms codon encoding due to less over-fitting and the difference between the training and validation loss error is 0.008.

## Introduction

COVID-19 is the most important pandemic of the 21st century. It started in Wuhan, China, in December 2019. As of May 20, there have been more than 165 million confirmed COVID-19 cases and over 3.4 million deaths worldwide (Chakraborty & Parvez, 2020). In February 2020, it has been considered a pandemic by the World Health Organization (WHO) due to its global impact. Severe acute respiratory syndrome coronavirus 2 (SARS-CoV-2) causes COVID-19 disease, and it is similar to other coronaviruses that have appeared in the past 2 decades, namely, the Middle East respiratory syndrome coronavirus (MERS-CoV) and severe acute respiratory distress syndrome coronavirus (SARS-CoV).

A large-scale search for powerful medications effective against the COVID-19 coronavirus has been undertaken but to date has not produced any results. Therefore, it is necessary to develop a vaccine because vaccination is an efficient approach to stop the pandemic with 95% effectiveness and is available to entire countries at the lowest cost. The vaccine activates the immune system of the body to identify and resist pathogenic agents such as bacteria, viruses and any related microorganisms (Khuroo et al., 2020). There are two vaccine designs: gene based and protein based. A protein-based vaccine teaches the immune system to fight the viruses or bacteria while the gene-based vaccines mimic the natural infection by holding the genetic instructions of the cell to generate the antigen, and particularly the surface spike protein in the case of coronaviruses (Abbasi, 2020). Many academic institutions and companies are working on COVID-19 vaccines with different strategies including mRNA in lipid nanoparticles, DNA, recombinant vectors, and proteins. Some vaccines such as mRNA have reached advanced phases testing; these vaccines encode the viral spike protein by mRNA as an antigen to elicit immune system response and produce neutralizing antibodies (Sempowski et al., 2020).

mRNA is one of the most promising and efficient approaches to the development of vaccines for COVID-19 and is based on the idea that the S protein in SARS-CoV can be used as the same mechanism to be applied on SARS-Cov-2 to build efficient vaccines against COVID-19 (Marian, 2021; Jackson et al., 2020). The mRNA-1273 vaccine is the first vaccine against COVID-19 (Jackson et al., 2020). It has many advantages such as an easy development process, low time required to develop, reduced risk of pre-existing immunity against the vaccine, speed, ease of automation, easy development of multiple prototype vaccines, and a less expensive process. Moreover, the greatest benefits of mRNA are safety and its non-infectious nature due to its direct injection and translation from messenger RNA into protein in the human body (Synced, 2020).

mRNA vaccines have become the leading candidates for immunization for COVID-19, but currently they face key potential restrictions. Designing stable mRNA molecules is currently the greatest challenge. It has been observed that RNA particles degrade suddenly; this is a significant limitation that means that the mRNA vaccine is useless if the mRNA suffers even one cut. Moreover, the mRNA vaccine for COVID-19 must be stored and transported under heavy refrigeration due to little knowledge of details where the backbone of RNA affected. Enhancing the stability of mRBA was a steep challenge in mRNA vaccine development prior to the pandemic, and the delivery of a refrigerator-stable vaccine against SARS-CoV-2 (COVID-19) is an important objective.

In this paper, we seek to use modern data science techniques, in particular, the deep learning paradigm, to design models and rules for predicting the degradation in mRNA molecules. The model will predict the likelihood of the degradation rates for each position inside the mRNA molecule. The model was trained on the Eterna dataset composed of 3000 mRNA sequences and structures and the scoring and predictions tested at Stanford University in parallel to our modeling (Stanford University, 2020).

The rest of this paper is organized as follows. A review of the literature for the latest research on COVID-19 is presented in “Literature Review”. Another part of the dataset used in this paper is described in “DataSet”. The proposed model architecture is described in detail in “Model Architecture”. The simulation and discussion are summarized in “Simulation and Discussions”, and the paper is concluded in “Conclusion and Future Works”.

## Literature Review

Vaccines save millions of lives each year, and according to the WHO, currently prevent 2–3 million deaths. There are currently over 169 COVID-19 vaccine candidates under development, with 26 of these in the human trial phase (World Health Organization (WHO), 2020).

Artificial intelligence and machine learning, particularly deep learning, have led to huge improvements in many fields of science and engineering because of their ability to learn features deeply. Vaccine discovery has been the most highly impacted area (Keshavarzi Arshadi et al., 2020). Recently, some powerful deep learning techniques as LSTM and GRU have been applied to the field of DNA and RNA sequence modeling. Artificial intelligence can be utilized to fight against COVID-19 pandemic and find a solution for the different areas such as drug discovery, vaccine development, public communications, and integrative medicine (Ahuja, Reddy & Marques, 2020).

Abbasi (2020) and Zhang et al. (2020) suggested that mRNA-based vaccines have emerged as a rapid and versatile platform to quickly respond to the challenge of COVID-19 pandemic and the success of mRNA vaccine had lead to the use of this approach in a variety of fields that are far away from COVID-19 to establish a broad platform for use against both other known and emerging pathogens.

Ong et al. (2020) tested some proteins for vaccine development against SARS and MERS. They used a machine learning Vaxign-ML reverse vaccinology tool and predicted six structural proteins with other five unstructured proteins to be adhesins that are crucial to the viral adhering and host invasion. They also provide a review of the current status of the coronavirus vaccine and found that there were only three SARS-CoV, six MERS-CoV and six SARS-CoV2 vaccine in clinical trials. Only one of these 15 vaccines was an mRNA-based vaccine (S protein) produced in the United Kingdom for SARS-CoV2, whereas Wang et al. (2020) reviewed 13 COVID-19 vaccines of which two are mRNA-based vaccines, the first developed by Moderna/NIH and the second by Pfizer/BioNTech, the vaccines candidates are mRNA1273 and BNT162b2. They mentioned that chemical modifications of the mRNA molecules may alter their proinflammatory activity, but the delivery vehicles and the mRNA condensing lipids can both induce unwanted proinflammatory responses.

Jackson et al. (2020) and Lu et al. (2020) conducted a phase1 open-label trial for mRNA-1273 vaccine on 45 healthy adults 18–55 years of age with different doses and concluded that the mRNA-1273 vaccine induced anti-SARS-CoV-2 immune responses in all participants, and no trial-limiting safety concerns were identified. On the other hand, Lu et al. (2020) mentioned that coronavirus virus-like particles assembly requires at least three structural proteins: S, M, and E and designed three mRNA vaccine candidates for COVID-19, and they encode various forms of antigens in vaccinated hosts.

Pfizer (New York, NY, USA) and BioNTech (Germany) introduced the BNT162b2 vaccine candidate based on mRNA. The vaccine candidate advanced to phase 2/3 study. They claim that their decision to select this candidate reflects the primary goal to bring a well-tolerated, highly effective vaccine to market as quickly as possible (Pfizer, 2020).

## Dataset

A Stanford COVID-19 mRNA vaccine dataset uploaded to Kaggle for competition is used as the primary dataset. It has two different types of sequence data and bpps data (Stanford University, 2020).

### Sequences data

The sequence data include two files for training and testing as shown in Table 1. The training file contains 19 fields, and the test file contains seven fields. The features fields are sequence, structure and predicted_loop_type; these fields describe the RNA sequence, whether a base is paired or unpaired and the structural context, respectively. The sequence is a combination of A, G, U, and C for each sample, and the structure is an array of (, ), and ‘.’. The predicted loop type field is a set of characters where each character in sequence as M: Multi-loop S: paired “Stem” B: Bulge I: Internal loop H: Hairpin loop X: eXternal loop E: dangling End. All of these fields have a length of 107 characters, but only 68 bases are scored.

**Table 1:** mRNA COVID-19 vaccine dataset.

File name	No. of samples	Sequences length	Sequences scored
Train	2,400	107	68
Augmented	2,400	107	68

The predicted fields are: reactivity which determines the likely secondary structure of the RNA, deg_Mg_pH10 and deg_pH10 that determine the probability of degradation at the base after incubating with/without magnesium at high pH (pH 10). deg_Mg_50C and deg_50C determine the probability of degradation at the base after incubating with/without magnesium at high temperature (50 degrees Celsius) (Stanford University, 2020).

### BPPS data

The bpps data contains 6,034 .npy files. The bpps data are symmetric square matrices pre-calculated for each sequence with the same length as the sequence. This matrix gives the probability that each pair of nucleotides in the RNA forms a base pair in the ensemble of RNA secondary structures so that it provides more robust and rich information about the structures than a single RNA secondary structure (World Health Organization (WHO), 2020; Keshavarzi Arshadi et al., 2020).

### Augmented data

Data augmentation is a highly important process for increasing the number of the training samples and overcoming the problem of over-fitting. In the COVID-19 dataset, we need to predict the sequence, structure, and loops. Many packages to carry out these tasks are available, such as ARNIE, which is an API library in python. ARNIE supports multiple secondary structure packages. The size of augmented data is 2,400 which equals the original data.

## Model Architecture

**Figure 1:**
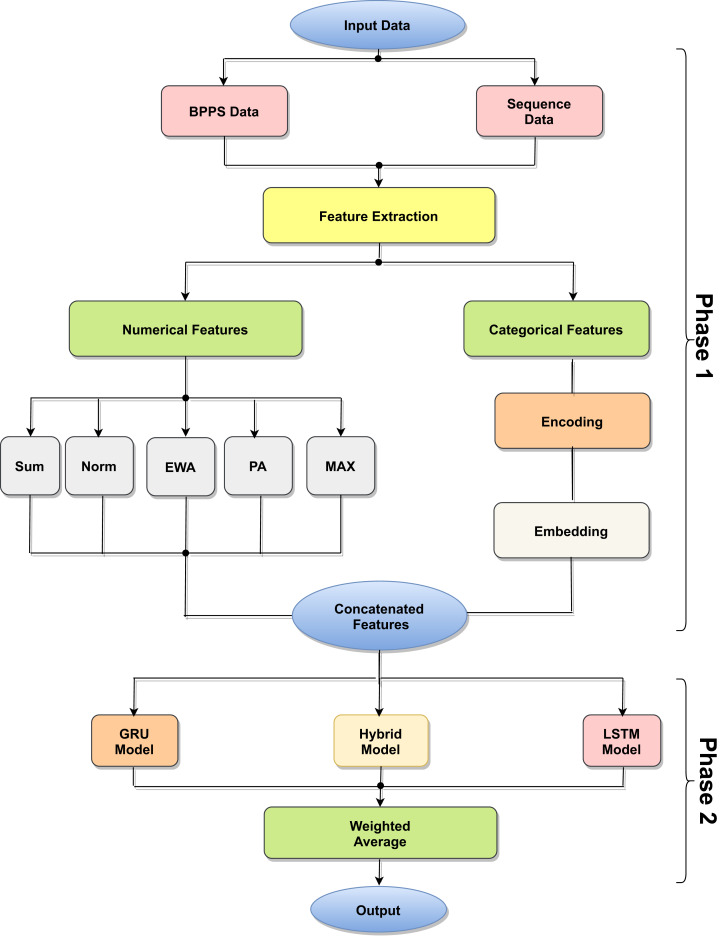
Model architecture process.

**Figure 2:**
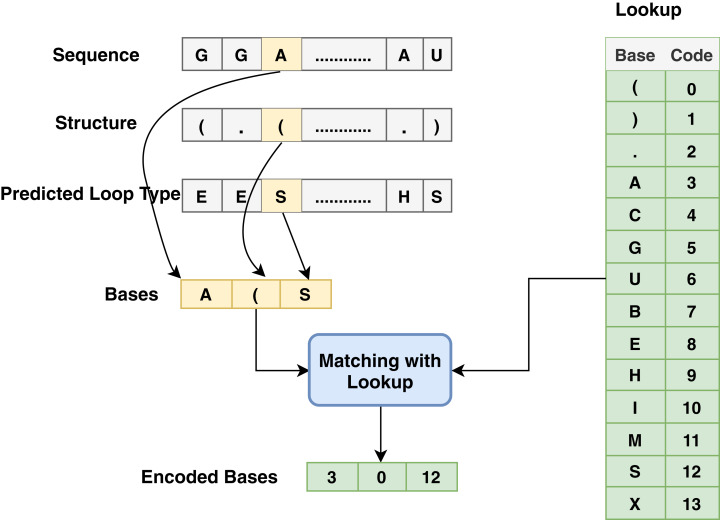
Base method steps for the encoding process associated with sequence, structure and predicted loop type data.

**Figure 3:**
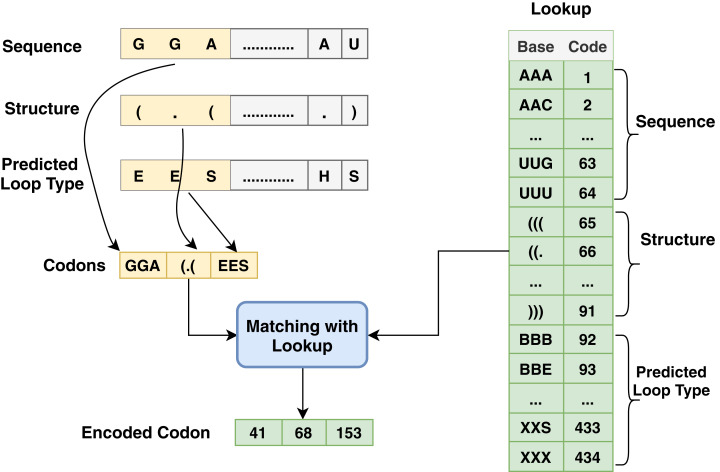
Codon method steps for encoding process associated with sequence, structure and predicted loop type data.

**Figure 4:**
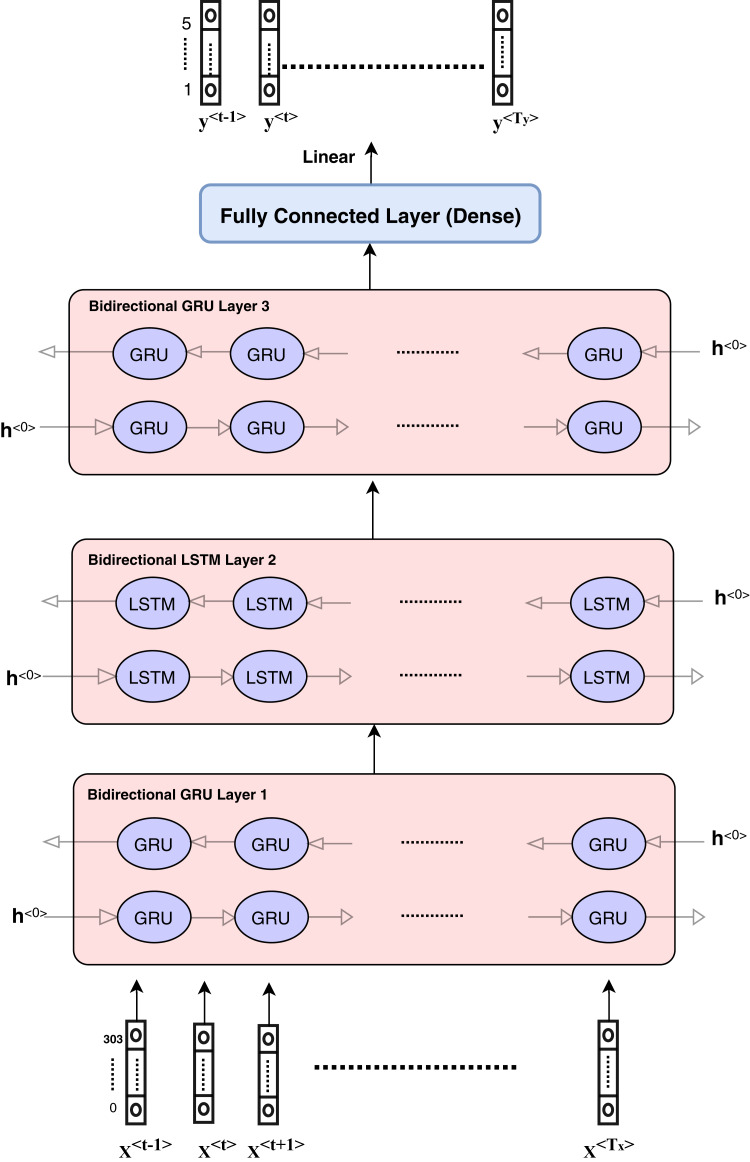
Architecture of hybrid bidirectional GRU and LSTM models.

The proposed model is based on sequence models. It is composed of bidirectional GRU, LSTM, and Hybrid models applied on mRNA sequence data and bpps data discussed under Dataset. The model first starts with feature engineering to extract the features and apply a sequence model to predict the mRNA sequences responsible for the degradation by predicting five reactivity values for every position in the sequence. The features engineering extracts two types of features called numerical features and categorical features. First, the categorical features are encoded, and an embedding layer is adopted to capture the relationships in the sequences that are very difficult to capture otherwise as shown in Fig. 1. Then, feature extraction is applied to extract numerical features using statistical and mathematical equations and categorical features as per shown in Figs. 2 and 3. Finally, the two types of features are concatenated to apply the bidirectional model as shown in Fig. 4.

### Features engineering

This work applies two approaches to discover and create the most important features based on the existing features to improve the predictive model performance. The following two sections explain the two types of features.

#### Numerical features

We tested many numerical formulae and the formulae selected based on the empirical results. All of the following formulae rely on the pre-calculated base pair probabilities in bpps matrices for each sequence. Every position in the sequence has a vector called bpp, which is a column in the bpps matrix. Here, n is the length of the bpp vector, and bppi is the probability of the given base with the position i for all the following formulae.

(1) }{}Sum = \sum\limits_{i = 1}^n bp{p_i}

(2) }{}Max = \max (bpp)

The following formula normalizes the average *μ* based on all bpps matrix values and standard deviation *σ*.

(3) }{}Norm = \displaystyle{{\bigg(\displaystyle{{\sum\nolimits_{i = 1}^n bp{p_i}} \over n}\bigg) - \mu } \over \sigma }

This formula is the exponential weighted average (EWA) for every vector of probabilities in the bpps matrix for a given base.

(4) }{}EWA = \sum\limits_{i = 1}^{n + 1} {V_i}

(5) }{}{V_i} = \beta {V_{i - 1}} + (1 - \beta )bp{p_{i - 1}}

where Vi is a weighted probability for every position i in the bpp vector and *β* is a constant value equal to 0.9

(6) }{}PA = \displaystyle{{\sum\nolimits_{i = 1}^n i*bpp(i)} \over n}

This feature describes the position multiplying the value of position in bpp to provide the position average value.

#### Categorical features

Since most machine learning techniques accept numerical features only, preprocessing and preparing the data to convert the categorical features into numerical features is a necessary step for enabling the technique to process the data and extract valuable information. In the mRNA vaccine data, there are three types of sequence data to be encoded using **base** method: sequence (that has AGUC characters) encoded from 3 to 6, structure (which has (). Characters) encoded from 0 to 2 and modified-loop (which has BEHIMSX characters) encoded from 7 to 13 as shown in Fig. 2.

Another type of encoding used in this work depends on codon-based encoding which used in many researches (Zhang et al., 2019; Hu et al., 2020) as an initial representation of the data and it provides deep learning techniques to extract high level features and capture the long dependency within the sequences. Each group of codon-based method has three bases in mRNA that constitutes a codon where each codon specifies a particular amino acid. The chain of amino acids forms a protein during mRNA translation. The sequence has AGUC characters and is encoded from 1 to 64, and the structure has (). characters and is encoded from 65 to 91; meanwhile, predicted-loop has BEHIMSX characters and is encoded from 92 to 434 as shown in Fig. 3.

After encoding, the resulting codes are fed into the embedding layer, which is a powerful feature that allows additional information to be automatically inserted into the neural network. Embedding layers are often used in natural language processing (NLP); however, they can be used in this work because we can insert a larger vector instead of the index values. Embedding layer is a dimension expansion that provides more information to the model. In this work, the embedding layer extracts 300 features instead of the 14 input encoding features, where the bases are represented by dense vectors that represent the projection of the base into a continuous vector space. The position of a base within the vector space is learned from the mRNA sequence and is based on the bases that surround the base when it is used.

### Sequence modeling

Sequence modeling, specifically recurrent neural networks (RNNs), is designed to utilize the structure data or sequence data. It has an inner state to read input sequence and allow RNNs to capture the interactions between the different elements through the mRNA sequences and others. There are two types of RNNs: long short term memory units (LSTM) and gated recurrent units (GRU). LSTM is a complex type of RNNs proposed by Hochreiter & Schmidhuber (1997) and can solve sequential complicated hierarchical decomposition problems that cannot be solved with RNNs. It is an effective way to capture long-term sequential dependencies. Moreover, it does not suffer from any optimization in contrast to simple recurrent networks (SRNs). GRU is a new technique proposed by Cho et al. (2014). It is similar to LSTM for capturing the dependencies with varied time scale, and also can solve the vanishing gradient problems by using the update gate and reset gate.

The proposed model architecture is described in Fig. 4 and consists of different layers and models. The first model is composed of three bidirectional GRU layers and is called the GRU model. The second model is composed of three bidirectional LSTM layers and is called the LSTM model. The third model is a hybrid model in which GRU and LSTM are used together as the bidirectional GRU layer, bidirectional GRU layer, and bidirectional LSTM layer, respectively. The output is weighted for all models as an ensemble model that provides better results.

The hybrid model consists of the GRU and LSTM units. After data pre-processing steps, encoding and embedding processes are prepared and described in the ‘Features engineering’ section, and the features are ready to enter the sequence modeling as inputs. Each sequence or sample includes input dimension as (*T*_*x*_,*Vector*_*Length*) such that *T*_*x*_ = 107 and *vector*_*length* depends on the number of numerical features and categorical features. The categorical features length equals to 300 and numerical features length is equal to 5.

The hidden units at each layer are 256 in each direction, which means that the number of bidirectional layer units is equal to 512. The bidirectional layer is used to optimize the results as the data are passed in the forward and backward directions to more flexibly capture the information in the sequence data. The hybrid model is presented in detail in Fig. 4. The GRU model can be described according to the layers and parameters in Table 2. Other models have the same architecture and parameters.

**Table 2:** GRU sequence model summary for layers, output shape and number of parameters in each layer (keras summary function).

Layer (type)	Output shape	Number of parameters
input_2 (Input Layer)	[(None, 107, 8)]	0
tf_op_layer_strided_slice_2	[(None, 107, 3)]	0
embedding (Embedding)	(None, 107, 3, 100)	1,400
tf_op_layer_Reshape (TensorFlow)	[(None, 107, 300)]	0
tf_op_layer_strided_slice_1(TF)	[(None, 107, 3)]	0
concatenate (Concatenate)	(None, 107, 305)	0
bidirectional (Bidirectional)	(None, 107, 512)	861,696
bidirectional_1 (Bidirectional)	(None, 107, 512)	1,182,720
bidirectional_2 (Bidirectional)	(None, 107, 512)	1,182,720
tf_op_layer_strided_slice_4	[(None, 68, 512)]	0
dense (Dense)	(None, 68, 5)	2,565
Total params: 3,231,101		
Trainable params: 3,231,101		
Non-trainable params: 0		

For each time t, *X*_*t*_ ∈ *R;*^*m*^^×^^*d*^ is the mini-batch input (number of observations: m, number of inputs: d) and *σ* is the hidden layer activation function. We suggest the forward and backward hidden states in the bidirectional architecture for this time step are }{}{\overrightarrow H _t} \in {\Re ^{m \times h}} and }{}{\overleftarrow H _t} \in {\Re ^{m \times h}} respectively. Here h refers to the number of hidden units. The forward and backward hidden state updates are computed as follows:

(7) }{}{\overrightarrow H _t} = \sigma ({X_t}W_{xh}^{(f)} + {\overrightarrow H _{t - 1}}W_{hh}^{(f)} + b_h^{(f)}),

(8) }{}{\overleftarrow H _t} = \sigma ({X_t}W_{xh}^{(b)} + {\overleftarrow H _{t + 1}}W_{hh}^{(b)} + b_h^{(b)}),

where the weight parameters }{}W_{xh}^{(f)} \in {\Re ^{d \times h}}, }{}W_{hh}^{(f)} \in {\Re ^{h \times h}}, }{}W_{xh}^{(b)} \in {\Re ^{d \times h}}, and }{}W_{hh}^{(b)} \in {\Re ^{h \times h}} and bias parameters }{}b_h^{(f)} \in {\Re ^{1 \times h}} and }{}b_h^{(b)} \in {\Re ^{1 \times h}} are all model parameters.

Then, the forward and backward hidden states are concatenated }{}{\overrightarrow H _t} and }{}{\overleftarrow H _t} to build the hidden state }{}H_t \in {\Re ^{n \times 2h}} and input it to the output layer. The data are transferred as inputs to the next bidirectional layer in deep bidirectional RNNs. Finally, }{}Y_t \in {\Re ^{m \times q}} (m: number of observations and q:number of outputs) is the output layer and is calculated as follows:

(9) }{}{Y_t} = {H_t}{W_{hq}} + {b_q},

where the model parameters are as follows: }{}W_{hq} \in {\Re ^{2h \times q}} is the weight parameter. }{}b_{q} \in {\Re ^{1 \times q}} is bias parameter of the output layer. The number of hidden units can be different in the bidirectional model.

Accordingly, the information from past and future is utilized to estimate the current state. This means that the output is estimated by the the information from both ends of the sequence which is the main feature in the bidirectional RNN. The global parameters that are used and configured in each bidirectional layers are summarized in Table 3.

**Table 3:** Bidirectional configuration parameters.

Parameter	Value
Hidden units	256
Dropout	0.4
Return sequences	True
Kernel initializer	‘orthogonal’

The dropout process is a regularization technique used to overcome the over-fitting problem in a learning process such that the model can generalize the results in stable manner with less error through testing and scoring in production. In this method, random neurons are dropped out and ignored during training while other neurons are used to represent the predictions. This makes the network less sensitive to the particular weights so that it can generalize better in testing and avoid the over-fitting problem.

Finally, the dense layer has five outputs and the activation function is linear because the problem is a regression problem. The optimizer is ‘Adam’, which is an efficient optimization method to update the weights in an efficient manner. It is used in deep learning due to its many advantages with respect to computation, memory, simplicity, and because it is appropriate for noisy data and non-stationary objectives. The loss function is MCRMSE as described in Eq. (10).

## Simulation and Discussions

The experiments are applied on the Stanford COVID-19 mRNA vaccine dataset as described in ‘Dataset’. The features are the combination of numerical features with length of 5 as described in ‘Numerical features’ and categorical features extracted from encoding and embedding processes, respectively, with embed_dim = 100 for each character from the sequence, structure, and predicted loop type and total concatenated length = 300 as described in ‘Categorial features’.

The experiments are performed in the GPU environment. The training data have 2,400 samples or observations. We filter the training data with the signal_to_noise feature to exclude the noisy samples that have values ≤ 1. Signal_to_noise is defined as mean (measurement value over 68 nts)/mean (statistical error in measurement value over 68 nts). Therefore, after applying the filter, there are 2096 training data. To reduce the complexity, we split the data into the training data containing 1886 samples for the construction of the model and the validation data containing 210 samples for the tuning and monitoring of the model, and to select the best model. The final size for the training data set after embedding is (1,886, 107, 8). The most important configuration of the model is: seq_len = 107, pred_len = 68, dropout = 0.5, embed_dim = 100, and hidden_dim = 256.

The model predicts five target columns of reactivity, deg_Mg_pH10, deg_pH10, deg_Mg_50C, and deg_50C and the measure of evaluation is the mean columnwise root mean squared error (MCRMSE). It is given by:

(10) }{}MCRMSE = \displaystyle{1 \over {{N_t}}}\sum\limits_{j = 1}^{{N_t}} \sqrt {\displaystyle{1 \over n}\sum\limits_{i = 1}^n {{({y_{ij}} - {{\hat y}_{ij}})}^2}}

where *N*_*t*_ is the number of scored ground truth target columns, and *y* and \hat y are the actual and predicted values, respectively.

**Figure 5:**
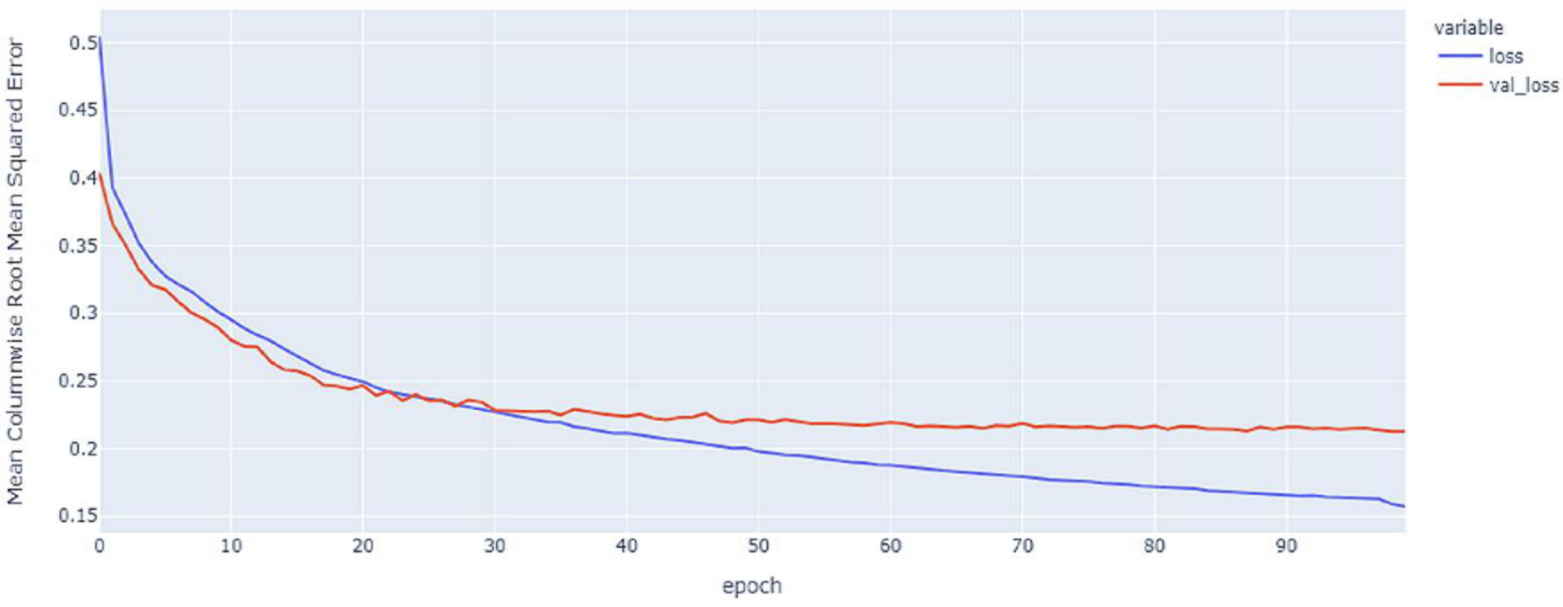
GRU model MCRMSE results on the categorical and numerical features without augmentation based on base encoding.

**Figure 6:**
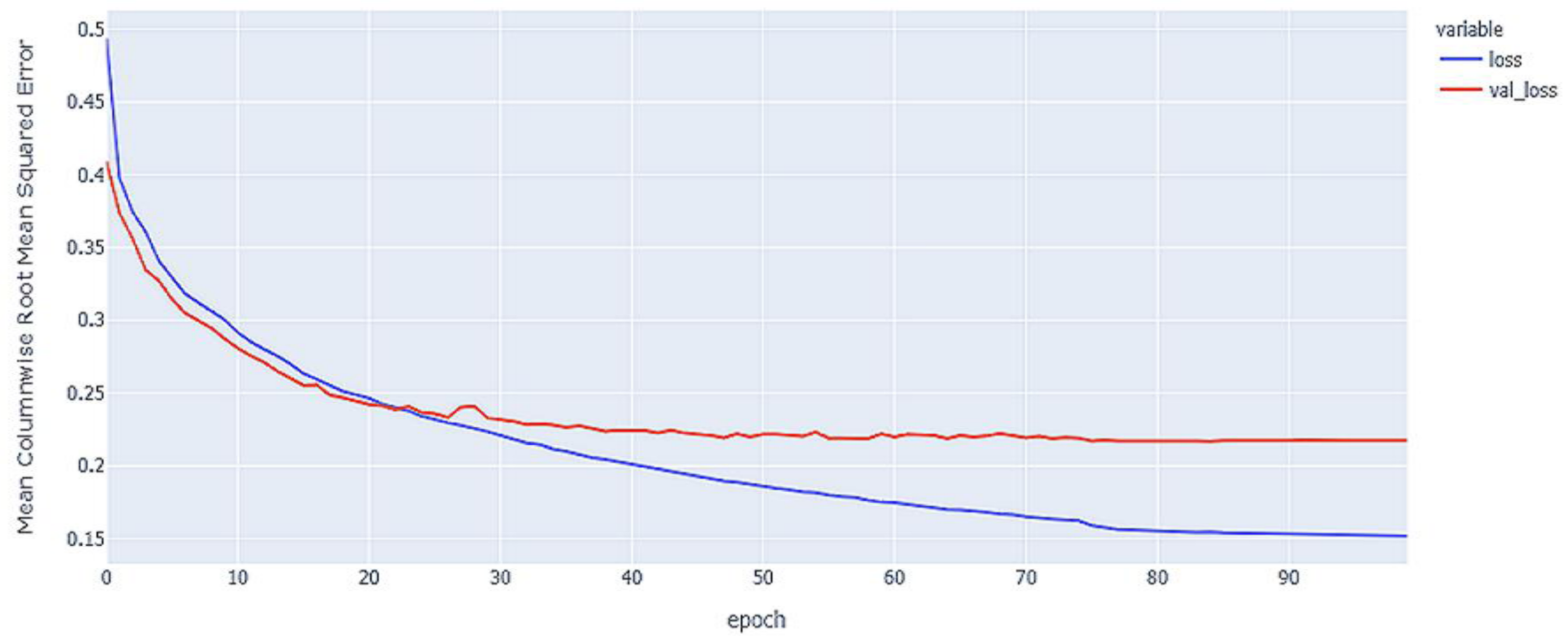
LSTM model MCRMSE results on the categorical and numerical features without augmentation based on base encoding.

**Figure 7:**
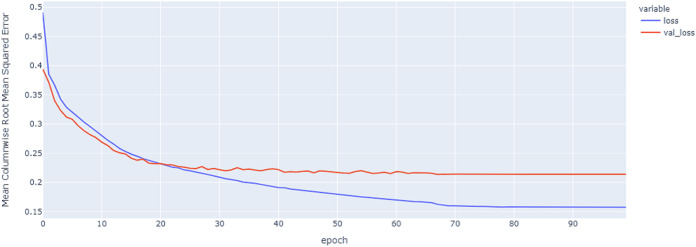
Hybrid LSTM model MCRMSE results on the categorical and numerical features without augmentation based on base encoding.

**Figure 8:**
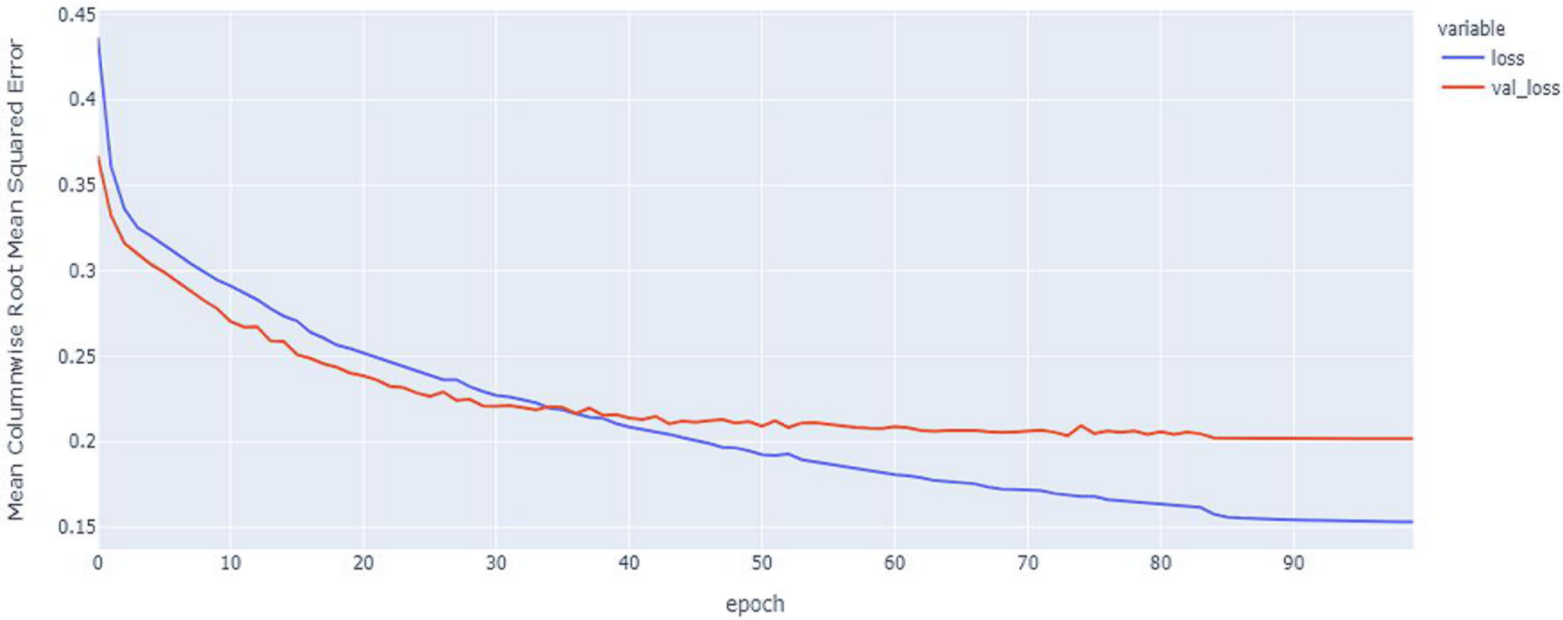
GRU model MCRMSE results on the categorical and numerical features without augmentation based on codon encoding.

**Figure 9:**
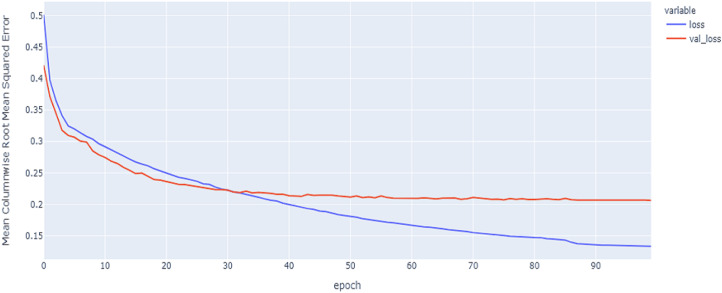
LSTM model MCRMSE results on the categorical and numerical features without augmentation based on codon encoding.

**Figure 10:**
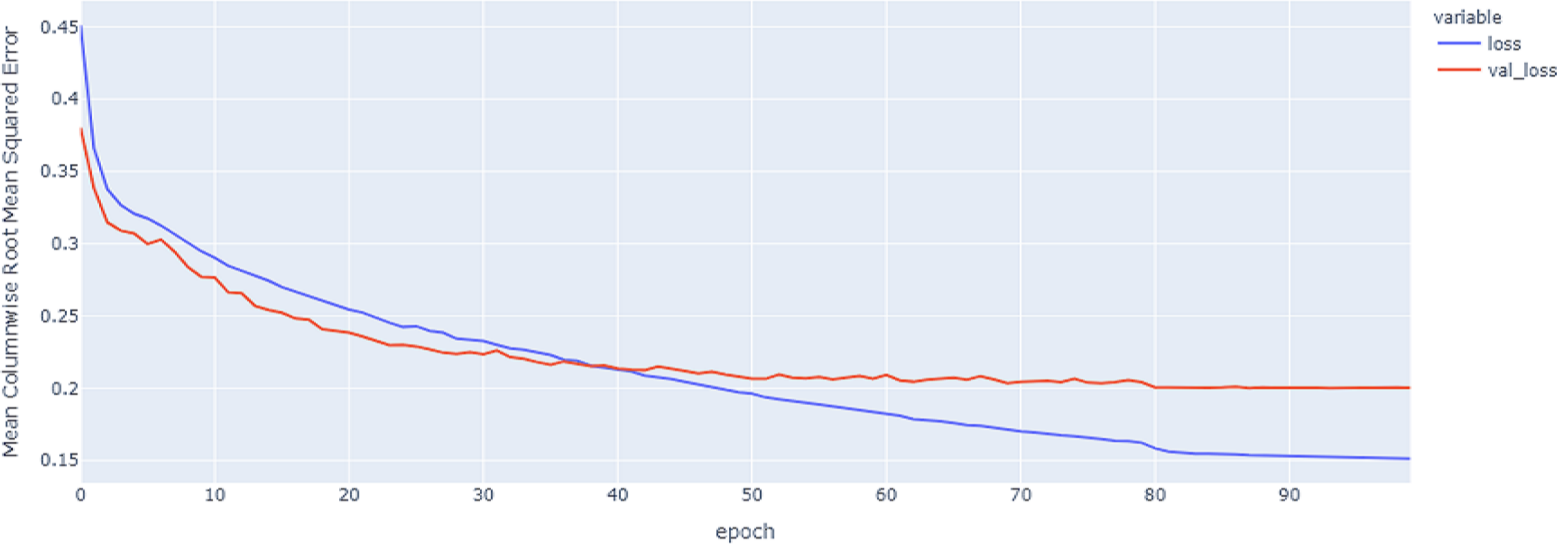
Hybrid model MCRMSE results on the categorical and numerical features without augmentation based on codon encoding.

As shown in Table 4 and Figs. 5, 6, 7, these results describe the change in MCRMSE with increasing number of epochs in our experiments using original data without augmentation and using Base method encoding and the GRU, LSTM, and Hybrid models. In Table 5 and Figs. 8, 9, 10, the MCRMSE are presented for the different number of epochs in our experiments using original data without augmentation and using codon method encoding and the GRU, LSTM, and Hybrid models.

**Table 4:** MCRMSE results of the sequence models based on the numerical and categorical features and base encoding method without augmentation.

Model name	Training data	Validation data
GRU	0.157	0.213
LSTM	0.152	0.217
HYBRID	0.157	0.214
Weighted average	0.155	0.215

**Table 5:** MCRMSE results of the sequence models based on the numerical and categorical features and codon encoding method without augmentation.

Model name	Training data	Validation data
GRU	0.158	0.201
LSTM	0.133	0.206
HYBRID	0.131	0.138
Weighted average	0.141	0.182

The previous experiments suffer from over-fitting, and there is a large difference between the training loss error and validation loss error. This over-fitting occurs when the model fits too well to the training set because of the increasing number of features comparing with the smaller number of samples. In this paper, two different approaches are used to solve this problem: the first approach is dropout regularization for reducing over-fitting and improving the generalization of deep neural networks. The network becomes less sensitive to the specific weights of neurons and becomes more capable of better generalization and is less likely to overfit the training data. The second approach used data augmentation to increase the number of samples. This approach increases or augments the diversity of the data so that at each training stage, the model encounters a different version of the original data.

**Figure 11:**
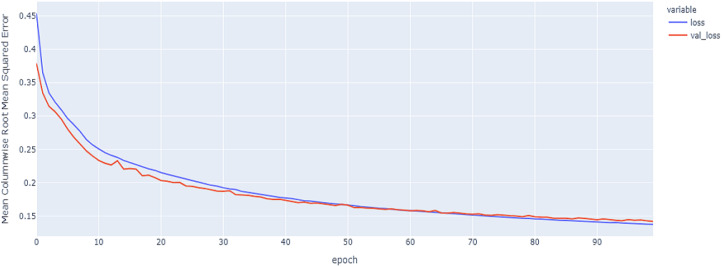
GRU model MCRMSE results on the categorical and numerical features and base encoding after augmentation.

**Figure 12:**
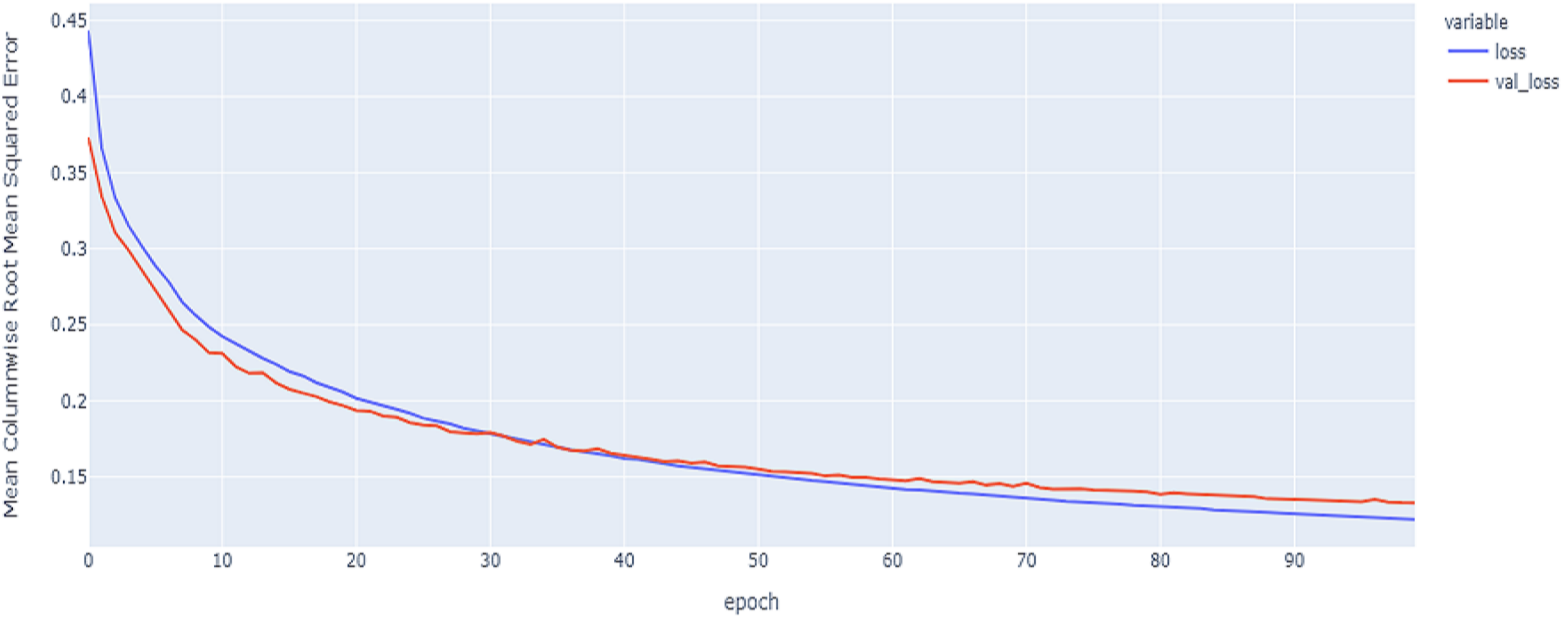
LSTM model MCRMSE results on the categorical and numerical features and base encoding after augmentation.

**Figure 13:**
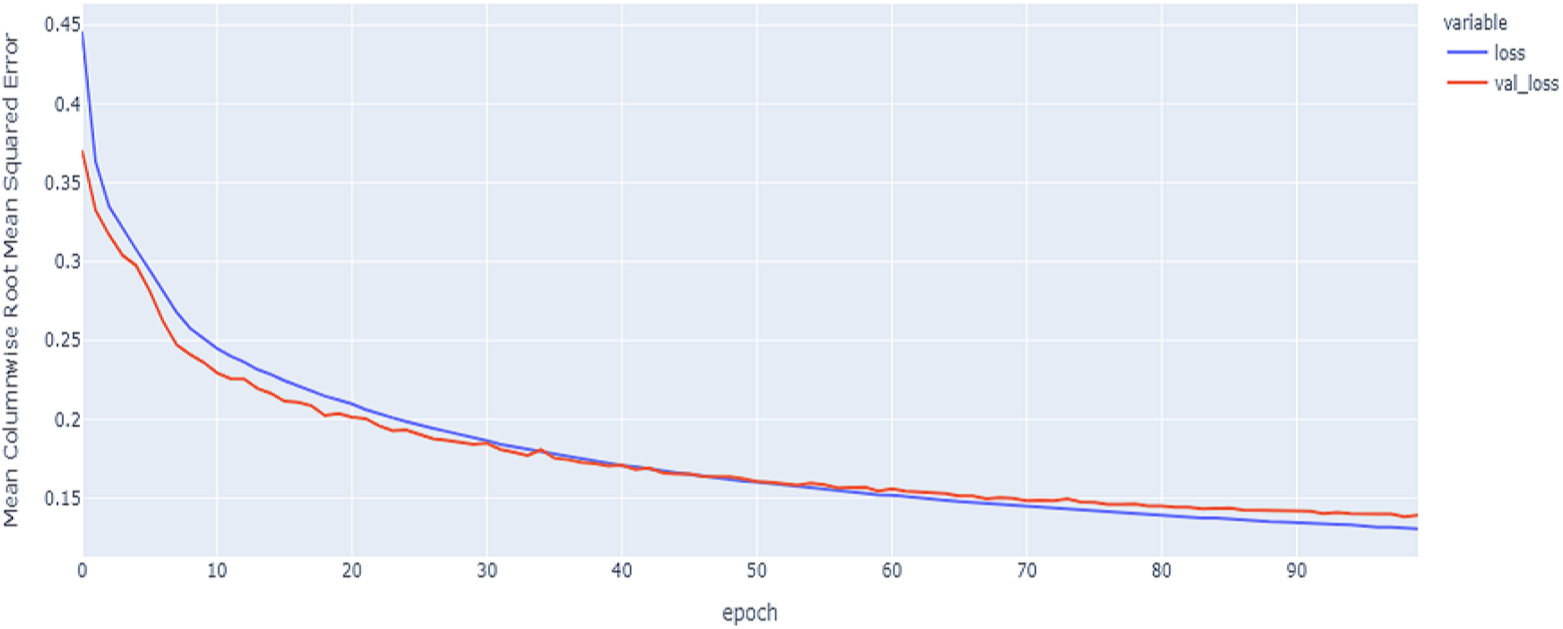
Hybrid model MCRMSE results on the categorical and numerical features and base encoding after augmentation.

**Figure 14:**
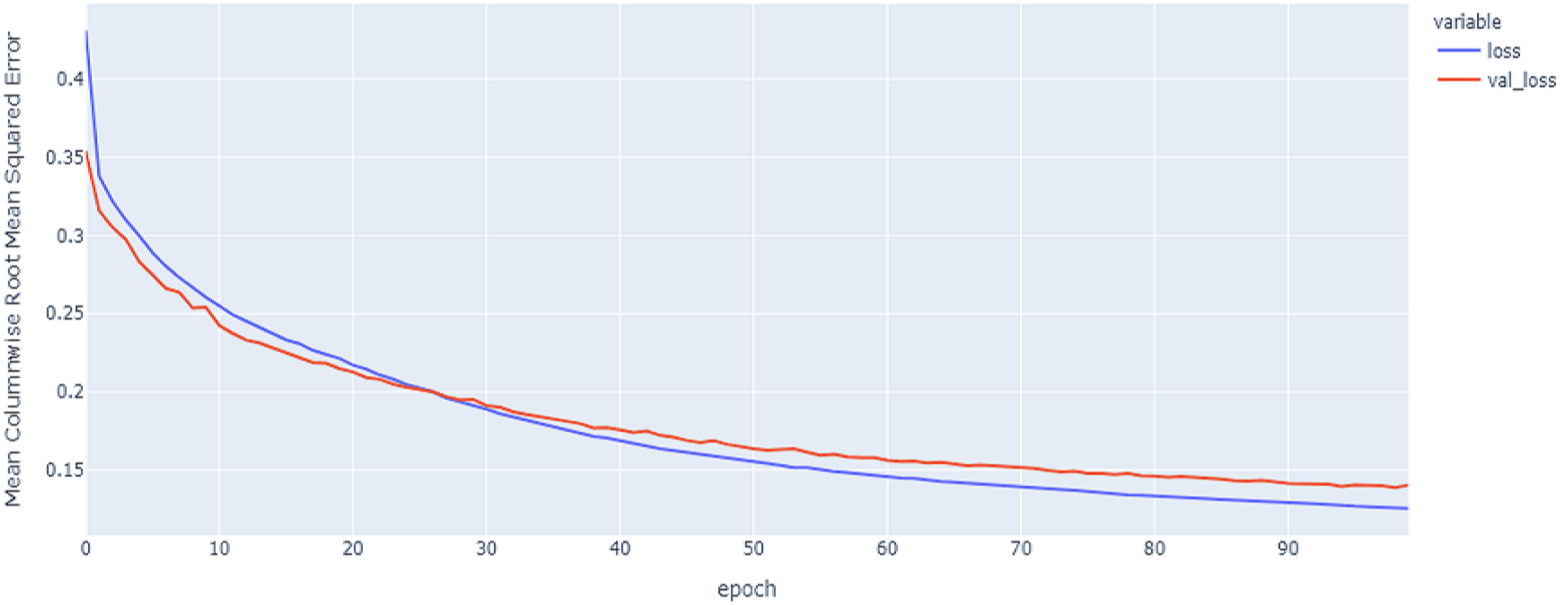
GRU model results on the categorical and numerical features with augmentation based on codon encoding.

**Figure 15:**
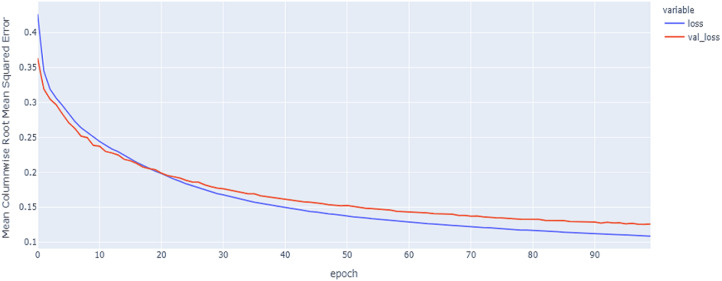
LSTM model results on the categorical and numerical features with augmentation based on codon encoding.

**Figure 16:**
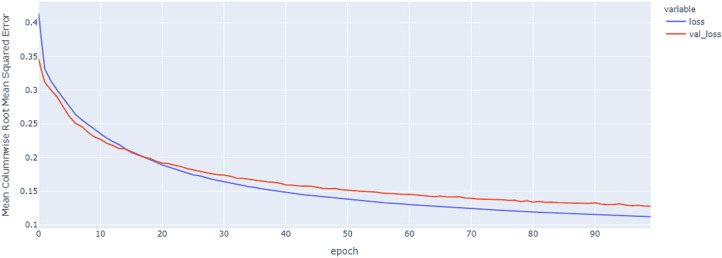
Hybrid model results on the categorical and numerical features with augmentation based on codon encoding.

In our implementations for data augmentation, we used the Vienna package to obtain the required data (tito:kaggle, 2020). The augmented data are concatenated with the original data to obtain 4,800 samples. The noisy data are excluded based on the signal_to_noise variable. Any sample that is ≤ 1 will be excluded. The training set size is 4,192. To reduce the complexity, we split the data into the training data with 3,772 samples to for the construction of the model and validation data with 420 samples to tune and monitor the models and select the best model. The final size for the training data after embedding is equal to: (3,772, 107, 8). As shown in Table 6 and Figs. 11, 12 and 13, the results of MCRMSE applied on e thGRU, LSTM, and Hyprid models based on Base encoding after augmentation and validation and training samples are closer and no over-fitting is observed. As shown in Table 7 and Figs. 14, 15 and 16, the results describe MCRMSE as a function of the number of epochs based on codon encoding after augmentation. Accordingly, augmentation resolved the issue and the model can generalize the prediction in production and real work.

**Table 6:** MCRMSE results of sequence models based on numerical and categorical features and base encoding after augmentation.

Model name	Training data	Validation data
GRU	0.138	0.142
LSTM	0.122	0.133
HYBRID	0.131	0.138
Weighted average	0.130	0.138

**Table 7:** MCRMSE results of sequence models based on the numerical and categorical features based on codon encoding method with augmentation.

Model name	Training data	Validation data
GRU	0.125	0.139
LSTM	0.109	0.125
HYBRID	0.112	0.128
Weighted average	0.115	0.131

The best model in our experiments is the LTSM model, which provides an MCRMSE of 0.125 using the codon encoding method and concatenated data of numerical and categorical features after augmentation. The best models for generalization with less over-fitting are mentioned in Table 6 with base encoding and after applying augmentation which the differences in MCRMSE are minimized between training loss error and validation loss error. The difference in MCRMSE is 0.008 using the weighted average of three sequence models. Accordingly, codon encoding outperforms base encoding with respect to MCRMSE validation error using LSTM model; meanwhile, base encoding outperforms codon encoding due to minimized MCRMSE and less over-fitting.

## Conclusion and Future Works

mRNA vaccines are the fastest vaccine candidates for the treatment of COVID-19 but currently are facing several limitations such as degradation. The sequence modeling approach uses sequence data as mRNA vaccine sequences. This paper used sequence modeling, GRU, LSTM and Hybrid for predicting mRNA sequences responsible for the degradation of COVID-19 mRNA vaccine. The sequence models predict degradation by predicting five reactivity values for every position in the sequence. We applied two encoding methods called base encoding and codon encoding methods. Moreover, we used augmentation techniques to generate new data using the ARNIE package that resolved the issue of over-fitting in our implementation to increase the number of samples and make the model more generalized in the prediction of new data. In another method to resolve the over-fitting, we configured the dropout regularization method in the bidirectional layers of GRU, LSTM, and Hybrid sequence models.

The model extracted 300 features using encoding and embedding layer extraction for the categorical features and five features as the numerical features. Then, the features are concatenated to the model. The models give promising results since they predict the five reactivity values with less validation MCRMSE of 0.125 using LSTM with codon encoding and after applying augmentation data. Meanwhile, the best models are presented with base encoding and augmentation with difference equals to 0.008 using weighted average of MCRMSE for three sequence models between training loss error and validation loss error; the results of training and validation loss errors are closer, and no over-fitting is obtained.

In the future, we plan to investigate more numerical features based on the characteristics of bioinformatics and RNA sequences to enhance the accuracy of the models and apply different classification models. Moreover, we also suggest applying a convolutional neural network (CNN) with sequence models to build a hybrid model with new features to improve the predictions. Graph convolutional networks (GCNs) have the ability to represent learning on a graph with the advantage of stacking deeper layers; therefore, this approach can be applied to evaluating mRNA degradation to get better results.

## Supplemental Information

10.7717/peerj-cs.597/supp-1 Supplemental Information 1 Train File.

10.7717/peerj-cs.597/supp-2 Supplemental Information 2 Code.

10.7717/peerj-cs.597/supp-3 Supplemental Information 3 Data Augmentation.

10.7717/peerj-cs.597/supp-4 Supplemental Information 4 Source Code.

## Additional Information and Declarations

### Data Availability

The following information was supplied regarding data availability: The scripts are available in the Supplemental Files. The data are available at Kaggle: https://www.kaggle.com/c/stanford-covid-vaccine/data.
